# Awareness of cancer symptoms and anticipated help seeking among ethnic minority groups in England

**DOI:** 10.1038/sj.bjc.6605387

**Published:** 2009-12-03

**Authors:** J Waller, K Robb, S Stubbings, A Ramirez, U Macleod, J Austoker, S Hiom, J Wardle

**Affiliations:** 1 Cancer Research UK Health Behaviour Research Centre, Department of Epidemiology and Public Health, UCL, Gower Street, London WC1E 6BT, UK; 2 Cancer Research UK Promoting Early Presentation Group, Institute of Psychiatry, King's College London, St Thomas' Hospital, London, UK; 3 General Practice and Primary Care, Division of Community Based Sciences, Faculty of Medicine, University of Glasgow, 1 Horselethill Road, Glasgow, UK; 4 Cancer Research UK Primary Care Education Research Group, Cancer Epidemiology Unit, University of Oxford, Richard Doll Building, Roosevelt Drive, Oxford, UK; 5 Health Information Department, Cancer Research UK, 61 Lincoln's Inn Fields, London, UK

**Keywords:** ethnicity, cancer awareness, cancer symptoms, anticipated delay, help seeking

## Abstract

**Objective:**

Little is known about ethnic differences in awareness of cancer-warning signs or help-seeking behaviour in Britain. As part of the National Awareness and Early Diagnosis Initiative (NAEDI), this study aimed to explore these factors as possible contributors to delay in cancer diagnosis.

**Methods:**

We used quota sampling to recruit 1500 men and women from the six largest minority ethnic groups in England (Indian, Pakistani, Bangladeshi, Caribbean, African and Chinese). In face-to-face interviews, participants completed the newly developed cancer awareness measure (CAM), which includes questions about warning signs for cancer, speed of consultation for possible cancer symptoms and barriers to help seeking.

**Results:**

Awareness of warning signs was low across all ethnic groups, especially using the open-ended (recall) question format, with lowest awareness in the African group. Women identified more emotional barriers and men more practical barriers to help seeking, with considerable ethnic variation. Anticipated delay in help seeking was higher in individuals who identified fewer warning signs and more barriers.

**Conclusions:**

The study suggests the need for culturally sensitive, community-based interventions to raise awareness and encourage early presentation.

The National Awareness and Early Diagnosis Initiative was set up in the United Kingdom to promote earlier diagnosis of cancer, with several work streams including one to assess public awareness of early signs of cancer and attitudes to help seeking and identify socio-demographic and cultural determinants of delay (Department of Health and Cancer Research UK, 2008).

In general, cancer incidence rates have been found to be lower in ethnic minority groups than in the general population in England (NCIN, 2009). However, there are notable exceptions to this, and rates of some cancers are rising to equal or exceed general population prevalence (Harding and Rosato, 1999; Smith *et al*, 2003; Wild *et al*, 2006). A recent report found, for example, that incidence of liver cancer is between 1.5 and 3 times higher in Asian groups than among whites, and incidence of prostate cancer is significantly higher in black men than white (NCIN, 2009). It should also be noted that cancer rates may seem lower than they are in some groups, because of lack of diagnosis, and high rates of other serious diseases, such as cardiovascular disease, that can affect people before the cancer is found (Wild and McKeigue, 1997; Wild *et al*, 2007).

Participation in the NHS cancer screening programmes also seems to be lower for breast (Hoare, 1996), cervical (Webb *et al*, 2004; Moser *et al*, 2009) and colorectal cancer in ethnic minority groups (Weller *et al*, 2007; Robb *et al*, 2008). These observations underline the importance of understanding attitudes towards cancer diagnosis in British ethnic minority populations, and this is mandated in the Race Relations (Amendment) Act (Office of Public Sector Information, 2000), which emphasises the need for racial equality in access to services.

The importance of cultural influences on recognition of symptoms and help-seeking behaviours was highlighted in a review of ethnic and cultural differences in models of and attitudes towards cancer across ethnic groups (Dein, 2004). The review found wide differences in beliefs both between countries, but also across ethnic and cultural groups within countries. Psychological factors such as fatalism, fear and embarrassment were identified as possible barriers to help seeking (Long, 1993; Frisby, 2002; Lannin *et al*, 2002), while lack of knowledge about cancer symptoms or early detection methods and misconceptions about causal processes also play a part (Gregg and Curry, 1994; Ratnasinghe *et al*, 1999). A recent qualitative study indicated that black and ethnic minority groups had poor knowledge about cancer, as well as beliefs and attitudes that might reduce attendance at services designed to promote early diagnosis (Thomas *et al*, 2005).

In the United States, African American women have higher death rates from breast cancer than white women, despite lower incidence levels (see Blackman and Masi, 2006), with patient delay explaining some of this disparity in outcome. Religiosity and spirituality have been suggested as possible explanatory factors, but recent research found educational level to be more important in explaining ethnic differences (Gullatte *et al*, 2009). Recent evidence from the Thames Cancer Registry suggests similar patterns in England. Black African and Black Caribbean women had lower age-standardised incidence rate ratios for breast cancer than white women, particularly for women aged 50 and over, but they were more likely to be diagnosed with metastatic disease and to die of it. Survival remained poorer in Black African women compared with white women after adjusting for age, deprivation and stage (Jack *et al*, 2009). A possible explanation for delayed presentation in black and ethnic minority women is poorer knowledge of breast cancer symptoms and less practising of breast awareness compared with white women (Scanlon and Wood, 2005). Aside from a few such studies of breast cancer awareness, however, very little is known about ethnic differences in cancer knowledge in the United Kingdom.

Stimulated by the neglect of research into awareness of, or attitudes towards, help seeking for cancer symptoms in ethnic minorities in England, this study used a new, validated measure to assess awareness of symptoms and help-seeking attitudes in the largest ethnic minority groups in England.

## Materials and methods

### Participants and methods

We commissioned an agency specialising in ethnic minority research (Ethnibus) to carry out the fieldwork. Quota sampling was used to recruit a total of 1500 participants from the six largest ethnic minority groups in the United Kingdom (Indian, Pakistani, Bangladeshi, Caribbean, African and Chinese) in proportion to their representation in the UK population. Additional quotas were used to ensure the inclusion of equal numbers of men and women and representation across age groups. Data were collected in two waves, carried out in October and November 2008. Postal areas in England with a high density of residents from each target ethnic group were randomly selected using 2001 census data. Multilingual interviewers visited households in the selected postal areas, and eligible individuals were invited to participate in face-to-face interviews in their preferred language. The instrument has not yet been formally translated, but the experienced interviewers were able to translate it during the interviews, as required. Recruitment continued until the quotas had been met. Participants were paid a small incentive of £5 to participate. Response rates were 48% and 56% in the October and November waves, respectively.

### Measures

The cancer awareness measure (CAM), development and details of which are described elsewhere (Stubbings *et al*, 2009), was used to assess awareness of the early warning signs of cancer, the speed with which people would contact their doctor to discuss each of nine possible cancer symptoms listed in Cancer Research UK's leaflet ‘Cancer – know the warning signs’ (see http://publications.cancerresearchuk.org/WebRoot/ crukstoredb/CRUK_PDFs/RTR200.pdf; we combined ‘changes in bowel and bladder habits’ to count as one sign, and separated ‘hoarseness/cough’ from ‘difficulty swallowing’) (see 2), and the importance of potential barriers to timely presentation. Awareness of warning signs was assessed using open questions (to index recall) and closed questions (to index recognition). Both methods have inbuilt biases: recall depends on memory and perseverance and probably underestimates knowledge, while recognition encourages guessing and over-estimates knowledge. Barriers included those in the ‘emotional’ domain (e.g. ‘I would be too embarrassed’), the ‘practical’ domain (e.g. ‘I would be too busy to make time to go to the doctor’) and the ‘service’ domain (e.g. ‘I would be worried about wasting the doctor's time’) (listed in 5). Warning sign awareness by either method was not only recorded sign by sign, but also totalled. Likewise, barriers were examined individually and the total was used to explore associations with anticipated delay. Anticipated delay in presentation was examined for each symptom and a total score calculated to quantify the number of symptoms for which participants would wait 2 weeks or more before contacting their doctor.

Socio-economic class (SEC) was assessed using the occupation of the chief earner in the household and was coded: AB, managerial/professional; C1, supervisory; C2, skilled manual; D, semi-skilled/unskilled manual and E, state pensioners or casual/lowest grade workers. These groupings are commonly used in market research (Meier and Moy, 2004). Age group, gender, ethnicity and main language spoken at home were also assessed.

### Analysis

Analyses were carried out using SPSS 14.0. The *χ*^2^ tests were used to examine ethnic differences in recall and recognition of each symptom, anticipated delay in help seeking and endorsement of barriers. ANCOVAs were used to examine predictors of scores for symptom awareness and anticipated delay in help seeking.

## Results

### Demographic characteristics

The demographic characteristics of the sample are shown by ethnic group in Table 1. The quotas for the six ethnic minority groups were met, with an even split by gender, and a good range across age and SEC groups. All the Caribbean and 75% of the African respondents reported speaking mainly English at home. This figure was much lower in the other groups: 30% of Indians, 24% of Bangladeshis, 21% of Chinese and 16% of Pakistanis. Other commonly spoken languages were Mandarin (63% of the Chinese group), Punjabi (28% of the Indian group and 56% of the Pakistani group), Urdu, Sylheti, Bangla, Hindi and Gujarati.

### Awareness of warning signs

Responses to the open and closed questions about warning signs are shown separately for each ethnic group in Table 2. In the unprompted (recall) format, a lump or swelling was the most commonly cited symptom, mentioned by 45–53% of respondents across the groups. The other symptoms were mentioned by far fewer people, with <10% in any group able to name a change in bowel/bladder habits, difficulty swallowing or a sore that does not heal. The *χ*^2^ tests were used to examine differences between groups for each symptom, except the three symptoms just mentioned for which too many cells had expected values of <5. There were no significant differences in recall of a lump/swelling, but all the other symptoms differed significantly between groups (*P*<0.01 for each). The mean number of symptoms cited in this format was 1.2 (95% confidence interval (CI): 1.1–1.2). The Caribbean group named most (1.5) and the African group fewest (1.0), with significant between-group differences (F(5,1494)=10.1, *P*<0.0001).

Responses to the prompted (recognition) questions about warning signs are also shown in Table 2. As expected, recognition was higher than recall. Again, a lump or swelling was the most-recognised symptom, with over 60% endorsement across the groups. However, unexplained pain was the most-endorsed symptom for the Bangladeshi (75%) and Caribbean (85%) groups. There were marked variations between groups for some symptoms, for example only around 40% of the Indian and Pakistani groups recognised a change in bowel/bladder habits, compared with 64% of Bangladeshis and 73% of Caribbeans. Ethnic differences were significant for all symptoms except cough/hoarseness (*P*<0.01 for all *χ*^2^ tests). The mean number of symptoms recognised was 4.7 (95% CI: 4.6–4.8) and as with the open question, the African group had the lowest score (4.3) and the Caribbean group the highest score (5.5). Analysis of variance showed a significant between-group difference (F(5,1494)=14.7, *P*<0.0001) in total recognition score.

Scores on the recognition and recall measures were significantly correlated overall (*r*=0.34, *P*<0.0001).

### Predictors of warning sign awareness

Analysis of covariance was used to examine independent demographic predictors of the total number of warning signs recalled or recognised (out of a possible nine). Estimated marginal means and significance values are shown in Table 3. For both recall and recognition, ethnicity and language were significantly associated with warning sign score, and age was associated with recognition, but not recall (higher recognition in older groups). SEC was dichotomised for this analysis and those in the higher group (ABC1) showed higher recall and recognition compared with those in the lower group (C2DE). There were no sex differences for either measure.

### Anticipated delay in help seeking

For each warning sign, we divided people into those who would contact their doctor in <2 weeks to discuss the symptom, and those who would wait 2 weeks or longer (see Table 4). For all ethnic groups, unexplained bleeding was associated with the least anticipated delay (only 4–21% said they would wait 2 weeks or more), and unexplained weight loss had the highest anticipated delay (73–87%). The *χ*^2^ tests showed significant differences between ethnic groups for all nine symptoms (*P*<0.05 in every case), with African and Caribbean groups anticipating the least delay. It is noteworthy that 56% of the Chinese group said they would wait at least 2 weeks before consulting their doctor about a lump or swelling, compared with <35% in each of the other groups.

### Barriers to help seeking

We asked respondents whether they would be put off going to the doctor by a series of emotional, practical and service barriers. For each barrier we divided people into those who responded ‘yes sometimes’ or ‘yes often’, and those who responded ‘no’ or ‘don’t know’ (see Table 5). The most frequently endorsed barrier was worry about what the doctor might find (41%), followed by difficulty making an appointment (40%) and having too many other things to worry about (37%).

Percentages for men and women in each ethnic group are displayed in Table 5. The *χ*^2^ tests revealed that overall, there were significant gender differences. Women were more likely than men to endorse all the emotional barriers, to be worried about wasting the doctor's time, to have too many other things to worry about and to find the doctor difficult to talk to (*P*<0.05). Endorsement of transport and appointment availability concerns was the same for men and women, but men were more likely to say that they would be too busy to make time to see the doctor (*P*=0.02) (although in the African group, women were more likely to endorse this barrier).

Differences between ethnic groups were significant for all barriers (*P*<0.01). The African group had the lowest endorsement of almost all the barriers, but other patterns were mixed. Within some ethnic groups, gender differences were marked – for example 32% of Bangladeshi men endorsed embarrassment as a barrier, compared with 82% of Bangladeshi women. When we summed the total number of barriers endorsed by each participant, mean score was highest for the Chinese group (3.5; 95% CI: 3.0–4.0) and lowest in the African group (1.6; 95% CI: 1.4–1.9). Differences across ethnic groups were significant (F(5,1494)=18.6, *P*<0.0001).

### Predictors of anticipated delay in help seeking

We created a scale of anticipated help-seeking delay by allocating a point for each symptom for which people said they would take 2 weeks or more to contact their doctor, which produced a scale with a range of 0–9. The overall mean score was 4.7 (95% CI: 4.6–4.8). There were significant differences between ethnic groups (F(5,1494)=24.8, *P*<0.0001). Scores were highest for the Bangladeshi and Chinese groups and lowest for the African group (see Table 4).

Analysis of covariance was used to examine predictors of anticipated delay. Ethnicity, language (English *vs* non-English) and SEC (ABC1 *vs* C2DE) were entered as categorical variables, with total number of warning signs recognised and total number of barriers as continuous variables. Recognition of warning signs (F(1,1476)=25.5, *P*<0.0001) and barriers score (F(1,1476)=29.5, *P*<0.0001) were significantly associated with anticipated delay, as was ethnic group (F(5,1476)=17.2, *P*<0.001). SEC and language did not have significant independent effects.

## Discussion

This is the first study to explore ethnic differences in awareness of cancer warning signs and beliefs about help seeking in a large sample in England. The use of quota sampling enabled us to collect a large enough sample to explore differences between ethnic groups, rather than simply comparing all ethnic minority groups with the white population, as is the tendency in population-based surveys. We were also able to examine associations between ethnicity and other demographic variables. The study explored awareness of the warning signs for cancer, anticipated help-seeking behaviour, and barriers to prompt help seeking, as well as associations between these variables.

Awareness of warning signs was low, especially in the open-ended (recall) format where a swelling or lump was the only symptom that was mentioned, unprompted, by more than half of respondents. Even in the recognition format, most respondents were only able to recognise four or five of the nine warning signs. The findings are strikingly different from those obtained in the UK population-based survey using the CAM, carried out at the same time (Robb *et al*, 2009), where 94% recognised a lump/swelling compared with 72% in this sample.

Differences between ethnic groups were significant and highlight particular gaps in awareness – for example only around 60% of Bangladeshis recognised a lump as a warning sign, whereas awareness was high (over 80%) in the Caribbean group. This may, in part, reflect differences in cancer incidence – the age-standardised breast cancer incidence rate ratio for Bangladeshi women compared with white women in a recent UK study was 0.23, which suggests that breast cancer symptoms may be less salient in this group (Jack *et al*, 2009). The figure for Caribbean women was much higher at 0.8. In contrast, 65% of Bangladeshis recognised bleeding as a symptom of cancer, compared with just 45% of the African group. This suggests that cognitive models of cancer, and especially understanding of its symptoms, may vary between ethnic groups, possibly because of variations in the incidence of different cancers, and points to the need for more in-depth explorations of the way in which cancer is conceptualised in different communities. It also highlights the importance of health professionals eliciting and taking into account their patients’ explanatory models of cancer, as suggested by Dein (2004).

Language had a significant independent association with symptom awareness, with poorer knowledge among those for whom English was not the main language spoken at home. This suggests the need for better provision of information to non-English speakers and is consistent with previous research calling for community-based information interventions for black and ethnic minority groups in the United Kingdom (Thomas *et al*, 2005).

Consistent with the population-based data (Robb *et al*, 2009), both recall and recognition of warning signs was found to be lower in lower SEC groups, and this effect was independent of the effect of ethnicity. However, it should be noted that total number of signs recalled and recognised in this study was lower than even the lowest social class group in the population representative sample (1.2 signs recalled in this study compared with 1.5 in the lowest SES group; 4.7 signs recognised in this study compared with 6.3 in the lowest SES group in the population sample). This may be due in part to higher overall deprivation in the Ethnibus sample, but the findings suggest that there are serious ethnic disparities in knowledge that must be tackled urgently. More work is needed to disentangle the effects of deprivation and ethnic background and to gain a better understanding of the mechanisms involved.

The study also indicated that variations in delay between ethnic groups were not explained solely by awareness of symptoms. The African group showed poorest recognition of symptoms, but were nevertheless most likely to anticipate seeking help quickly across all nine symptoms. The bivariate correlation between symptom recognition and prompt help seeking did not reach statistical significance for either the African or Chinese groups, whereas there was a significant positive correlation for all the other groups (*P*<0.02). This suggests the existence of different cultural norms around help –seeking, and although raising symptom awareness will be important, additional steps will be needed to encourage help seeking, particularly in some communities. This is consistent with a study of breast cancer symptom delay in African and Caribbean women in London (Littlewood and Elias, 2000). In this study, women expected that their symptoms would be diagnosed as cancer, but their fear of being rejected by their community because they had cancer led them to delay seeking help. More work is needed to better understand these kinds of culturally specific motivations.

Interestingly, this sample anticipated more delay overall than the general population sample, even though in their analysis, Robb *et al* (2009) found lower anticipated delay in people from non-white ethnic backgrounds. This reinforces the need for studies that are specifically designed to explore ethnic differences, as the findings from population-based surveys, where all non-white groups are combined for analysis, may be misleading. Our findings are consistent with a systematic review of factors associated with delayed presentation in patients diagnosed with breast cancer, which concluded that there was ‘moderate’ evidence for increased delay in non-white ethnic groups (Ramirez *et al*, 1999).

Our exploration of barriers to contacting the doctor sheds some light on possible explanations for the ethnic variations in help seeking. The African respondents were least likely to endorse the majority of barriers in the emotional, practical and service domains, with few differences between men and women, and this is consistent with their low anticipated delay. Embarrassment seemed to be a particularly important barrier among Bangladeshi and Pakistani women, while being worried about what the doctor might find was a problem for Indian, Caribbean and Chinese women, as well as Chinese men. Overall, concern about what the doctor might find was the most frequently endorsed barrier, followed by problems making appointments. About 40% of participants in this study and a very similar proportion of the general population sample described by Robb *et al* (2009) agreed that it would be difficult for them to arrange an appointment with their doctor.

Different barriers point to different interventions to encourage prompt help seeking. Concerns about what the doctor might find could be addressed with information interventions emphasising the benefits of early diagnosis and the efficacy of treatment for many cancers. Increasing the availability and convenience of GP appointments should help to overcome service provision barriers. Embarrassment may reflect concerns about being able to see a female doctor or arise from past experience of not having been taken seriously. Research with health professionals has found that many feel inadequately trained to deal sensitively with people from different ethnic backgrounds (Richardson *et al*, 2006), so additional support for health professionals in this area might be appropriate.

Once again, comparing these findings with the population-based data (Robb *et al*, 2009) reveals some marked differences. People in our ethnic minority sample were much more likely to believe that the doctor would be difficult to talk to (27% compared with 13% in the population sample) and to lack confidence in talking about their symptoms (30% compared with 12%). In contrast, the population-based sample was more likely to be concerned about wasting the doctor's time (38% compared with 28% in our sample). This underlines the need for culturally sensitive, targeted interventions to address barriers in different groups, and points to the importance of communication issues for ethnic minority groups, even where language *per se* is not a barrier.

### Strengths and limitations

The use of quota sampling, rather than random probability sampling, means that we cannot be sure that our respondents were representative of the ethnic groups from which they were drawn. This may be a particular problem for the African group, which is likely to have comprised people from a wide range of very different communities. However, this method of recruitment did achieve a sample size that allowed us to make between-group comparisons, which is rarely possible in population-based surveys because ethnic minorities make up only around 10% of the UK population. A further strength of the study was that participants had the option to have the interview conducted in their own language, whereas the population-based survey was only offered in English. Given the differences in findings between the studies, it suggests that this study may have obtained the views of people who are normally excluded from this type of research. In addition, the study benefited from using a systematically developed and psychometrically validated instrument to assess cancer awareness and help-seeking beliefs.

The study focused on symptom awareness, but this is only one aspect of cancer prevention and early detection. Understanding that cancer can be asymptomatic is also important, especially in relation to participation in population-based screening and this needs to be explored in future studies. In addition, more work is needed to better understand the attitudes and beliefs underpinning ethnic differences in behaviour relating to cancer prevention and help seeking.

### Conclusions

This is the first study to examine ethnic differences in awareness and early presentation with cancer symptoms in a large, ethnically diverse sample. The study has identified low levels of awareness of cancer warning signs, a wide range of barriers to attendance and a high level of anticipated delay in seeking help for possible cancer symptoms. More work is needed to gain an understanding of the ethnic differences uncovered by this survey and to develop appropriate, culturally sensitive interventions to reduce ethnic disparities in knowledge and access to services.

## Conflict of interest

The authors declare no conflict of interest.

## Figures and Tables

**Table 1:** Demographic characteristics by ethnic group

	**Indian (*n*=467)**	**Pakistani (*n*=333)**	**Bangladeshi (*n*=126)**	**Caribbean (*n*=252)**	**African (*n*=216)**	**Chinese (*n*=106)**
	**(%)**	**(%)**	**(%)**	**(%)**	**(%)**	**(%)**
*Sex*						
Male	49.9	50.2	52.4	46.8	48.1	50.9
Female	50.1	49.8	47.6	53.2	51.9	49.1
						
*Age group*						
18–24	21.8	30.0	30.2	19.4	19.4	18.9
25–34	24.8	27.9	31.0	22.6	30.1	23.6
35–44	22.3	16.2	16.7	19.8	28.7	29.2
45–54	15.6	12.6	8.7	16.3	14.4	12.3
55+	15.4	13.2	13.5	21.8	7.4	16.0
						
*Language*						
English	30.4	16.2	23.8	100	75.0	20.8
						
*Social class*						
AB	13.1	7.8	8.7	6.7	15.3	17.0
C1	30.8	31.5	23.8	19.4	28.7	29.2
C2	23.8	23.7	19.0	17.5	19.4	31.1
D	24.8	27.3	37.3	34.5	22.2	14.2
E	7.5	9.6	11.1	21.8	14.4	8.5

**Table 2:** Awareness of warning signs by ethnic group

	**All groups (*n*=1500)**	**Indian (*n*=467)**	**Pakistani (*n*=333)**	**Bangladeshi (*n*=126)**	**Caribbean (*n*=252)**	**African (*n*=216)**	**Chinese (*n*=106)**	**Between group difference (*P*)**
*Recall of warning signs (%)*								
Unexplained lump or swelling	50.4	49.7	51.4	45.2	51.2	53.2	49.1	NS
Unexplained pain	20.1	18.8	15.3	20.6	34.1	15.7	15.1	<0.0001
Unexplained weight loss	16.1	9.4	14.7	11.1	30.6	15.7	21.7	<0.0001
Unexplained bleeding	14.5	10.9	20.4	9.5	24.2	3.2	17.0	<0.0001
Cough/hoarseness	6.8	7.5	8.4	2.4	4.8	3.7	15.1	0.001
Change in a mole	6.3	7.5	10.2	6.3	2.4	3.7	2.8	0.001
Change in bowel/bladder habits	2.4	1.9	2.4	3.2	3.6	0.9	3.8	a
Sore that does not heal	1.1	0.2	3.6	1.6	0	0.9	0	a
Difficulty swallowing	0.4	0.4	0.9	0	0	0.5	0	a
Total (mean number recalled)	1.18	1.06	1.27	1.00	1.51	0.98	1.25	<0.0001
								
*Recognition of warning signs (%)*								
Unexplained lump or swelling	72.2	70.2	72.4	61.1	81.0	73.6	69.8	0.002
Unexplained pain	72.1	69.6	68.2	75.4	84.9	63.0	79.2	<0.0001
Unexplained bleeding	59.9	56.5	58.6	65.1	73.0	45.4	70.8	<0.0001
Unexplained weight loss	57.9	52.9	50.8	61.9	71.8	56.9	67.0	<0.0001
Change in bowel/bladder habits	51.2	43.7	39.9	64.3	73.0	51.4	51.9	<0.0001
Change in a mole	46.1	48.4	46.5	58.7	48.8	29.2	47.2	<0.0001
Cough/hoarseness	42.3	42.8	39.0	45.2	41.3	38.9	55.7	NS
Difficulty swallowing	37.1	35.8	28.8	46.8	44.4	31.5	50.9	<0.0001
Sore that does not heal	34.9	29.1	42.9	42.9	27.0	38.9	35.8	<0.0001
Total (mean number recognised)	4.74	4.49	4.47	5.21	5.45	4.29	5.28	<0.0001

a Some cells had an expected count of <5 so *χ*^2^ tests could not be performed.

**Table 3:** Analysis of covariance showing demographic predictors of recall and recognition of nine warning signs of cancer

	**Number of warning signs recalled^a^**		**Number of warning signs recognised^a^**	
	**Estimated marginal mean (95% confidence interval)**	**Significance**	**Estimated marginal mean (95% confidence interval)**	**Significance**
*Ethnic group*				
Indian	1.12 (1.31–1.22)	F(5,1487)=9.03, *P*<0.0001	4.65 (4.47–4.84)	F(5,1487)=12.33, *P*<0.0001
Pakistani	1.38 (1.27–1.50)		4.77 (4.54–5.01)	
Bangladeshi	1.10 (0.92–1.28)		5.51 (5.15–5.86)	
Caribbean	1.42 (1.27–1.56)		5.24 (4.96–5.53)	
African	0.92 (0.79–1.06)		4.24 (3.96–4.51)	
Chinese	1.31 (1.12–1.50)		5.47 (5.09–5.85)	
				
*Language*				
English	1.33 (1.23–1.43)	F(1,1487)=12.49, *P*<0.0001	5.26 (5.07–5.44)	F(1,1487)=16.78, *P*<0.0001
Other	1.09 (1.00–1.17)		4.71 (4.54–4.87)	
				
*Age*				
18–24	1.15 (1.04–1.26)	F(4,1487)=1.93, *P*=0.10	4.80 (4.58–5.01)	F(4,1487)=6.97, *P*<0.0001
25–34	1.15 (1.05–1.26)		4.57 (4.36–4.77)	
35–44	1.32 (1.21–1.43)		5.15 (4.93–5.37)	
45–54	1.16 (1.02–1.30)		5.10 (4.83–5.37)	
55+	1.27 (1.13–1.41)		5.29 (5.02–5.56)	
				
*Sex*				
Female	1.22 (1.15–1.30)	F(1,1487)=0.35, *P*=0.55	5.00 (4.85–5.16)	F(1,1487)=0.18, *P*=0.68
Male	1.19 (1.12–1.27)		4.96 (4.80–5.11)	
				
*Social class*				
ABC1	1.28 (1.19–1.36)	F(1,1487)=6.12, *P*=0.01	5.10 (4.93–5.28)	F(1,1487)=5.45, *P*=0.02
C2DE	1.14 (1.07–1.22)		4.86 (4.72–5.00)	

a Adjusted for all other variables in the model.

**Table 4:** Ethnic differences in percentage of people reporting they would contact the doctor in <2 weeks to discuss a symptom and total delay score

**Warning signs**	**All groups (*n*=1500)**	**Indian (*n*=467)**	**Pakistani (*n*=333)**	**Bangladeshi (*n*=126)**	**Caribbean (*n*=252)**	**African (*n*=216)**	**Chinese (*n*=106)**	**Between group difference (*P*)**
Unexplained bleeding	87.0	81.8	87.7	88.1	90.5	96.3	79.2	<0.0001
Unexplained pain	78.7	73.2	75.7	77.8	86.9	92.1	66.0	<0.0001
Unexplained lump or swelling	74.4	68.3	75.1	65.1	86.5	92.6	44.3	<0.0001
Change in bowel/bladder habits	66.5	60.0	63.7	53.2	83.7	80.1	50.9	<0.0001
Sore that did not heal	61.4	57.8	70.0	53.2	50.4	79.2	50.0	<0.0001
Change in a mole	56.9	49.0	58.0	51.6	63.1	69.0	54.7	<0.0001
Difficulty swallowing	46.3	40.0	32.4	42.1	56.7	67.6	54.7	<0.0001
Cough/hoarseness	37.5	36.8	38.7	34.9	30.6	51.9	26.4	<0.0001
Unexplained weight loss	20.2	18.6	18.6	13.5	22.2	26.9	21.7	0.04
Total delay score (mean number of symptoms with at least 2 weeks delay)	4.7	5.1	4.8	5.2	4.3	3.4	5.5	<0.0001

**Table 5:** Reasons for putting off going to the doctor with a potentially serious symptom (% responding ‘yes sometimes’ or ‘yes often’) by sex and ethnic group

	**All groups**	**Indian**		**Pakistani**		**Bangladeshi**		**Caribbean**		**African**		**Chinese**	
	***n*=1500**	**Men *n*=233**	**Women *n*=234**	**Men *n*=167**	**Women *n*=166**	**Men *n*=66**	**Women *n*=60**	**Men *n*=118**	**Women *n*=134**	**Men *n*=104**	**Women *n*=112**	**Men *n*=54**	**Women *n*=52**
*Emotional barriers*													
Worried about what doctor might find	41.2	42.5	56.0	36.5	44.0	31.8	36.7	31.4	49.3	18.3	24.1	57.4	59.6
Too embarrassed	35.9	22.7	33.8	33.5	58.4	31.8	81.7	35.6	42.5	24.0	20.5	37.0	30.8
Too scared	32.4	26.6	30.3	35.9	49.4	16.7	25.0	29.7	45.5	25.0	21.4	40.7	32.7
Would not feel confident talking about symptom	29.7	32.2	30.8	20.4	36.7	24.2	33.3	37.3	39.6	17.3	21.4	24.1	30.8
													
*Practical barriers*													
Too many other things to worry about	36.7	37.8	49.1	37.7	44.0	40.9	53.3	28.8	25.4	13.5	19.6	46.3	46.2
Too busy to make time	35.4	45.1	37.2	38.9	35.5	43.9	31.7	35.6	25.4	16.3	21.4	50.0	44.2
Difficult to arrange transport	18.1	17.2	17.1	17.4	28.9	16.7	16.7	16.1	20.9	8.7	6.3	29.6	26.9
													
*Service barriers*													
Difficult to make an appointment	39.5	33.9	40.6	46.1	47.0	42.4	40.0	43.2	42.5	28.8	23.2	44.4	46.2
Worry about wasting doctor's time	27.5	20.6	41.9	28.1	39.2	25.8	40.0	25.4	27.6	8.7	9.8	25.9	23.1
Doctor would be difficult to talk to	26.6	21.5	26.9	24.6	34.3	30.3	41.7	28.0	20.1	12.5	20.5	38.9	50.8
